# Ruptured Anterior Inferior Cerebellar Artery Aneurysm From a Persistent Trigeminal Artery: A Case Report

**DOI:** 10.7759/cureus.60648

**Published:** 2024-05-20

**Authors:** Hasan Ahmad, Ahmed Albayar, Najib Muhammad, Dominic Romeo, Mohamed Salem, Andrew I Yang, Jan-Karl Burkhardt

**Affiliations:** 1 Department of Neurosurgery, Hospital of the University of Pennsylvania, Philadelphia, USA; 2 Department of Neurosurgery, Barrow Neurological Institute, Philadelphia, USA

**Keywords:** posterior circulation aneurysm, clipping, anterior inferior cerebellar artery, persistent trigeminal artery, aneurysm

## Abstract

Persistent trigeminal artery (PTA) is the most common remnant of the primitive carotid-vertebrobasilar anastomoses, which typically form and obliterate during the early stages of human embryonic development. While PTA can be non-pathologic and is usually an incidental finding, it is also associated with various other vascular abnormalities, such as arteriovenous malformations and fistulae, but most commonly cerebral aneurysms. In these cases, aneurysms are usually reported in the anterior cerebral circulation or in the PTA trunk itself; to date, only one report exists of an associated aneurysm in the posterior circulation (basilar artery). These associated vascular pathologies are not only a source of morbidity and mortality but can also complicate subsequent endovascular treatment due to different flow patterns and increased vessel tortuosity. In this case report, we present the first reported case of PTA-associated aneurysm in the anterior inferior cerebellar artery and its resulting impact on the endovascular treatment of this aneurysm.

## Introduction

Carotid-basilar anastomoses are embryonic communications between the precursors of the carotid and vertebrobasilar circulations that supply blood to the hindbrain. While these primitive vessels are only present for a short period of time, from the 3-4 mm embryonic stage to the 7-12 mm stage [1], some anastomoses can persist into the adult period. A persistent trigeminal artery (PTA), derived from the embryonic primitive trigeminal artery, is the most common persistent primitive carotid-vertebrobasilar anastomosis, with historical and contemporary studies placing its incidence at approximately 0.5-0.7% [2-4]. Since the first angiographic demonstration of PTA in 1950 by Sutton [5], numerous cases of PTA have been reported, often in association with other vascular abnormalities such as cerebral artery hypoplasia [6], arteriovenous malformations [7], carotid-cavernous fistulae [8], and cerebral aneurysms. The association between PTA and cerebral aneurysms has been especially controversial. Initial studies suggested that up to 32% of patients with PTA develop aneurysms [9], possibly due to the presence of structural defects in the walls of the cerebral arteries. However, recent reports suggest that this comorbid incidence ranges from 3% to 4% [3,10], which approximates the incidence of cerebral aneurysms in the general population [11]. The current literature is largely constrained to single case reports due to the low prevalence and incidental presentation of PTA. Among these published reports, aneurysms are almost exclusively in the anterior cerebral circulation, most commonly the posterior communicating artery (PCOM). Rarely, aneurysms can be found in the PTA trunk itself [12] or, as in one case describing a cerebral aneurysm in the basilar artery (BA) of a patient with PTA, in the posterior cerebral circulation [13]. Still, more work is needed to better quantify and characterize the association between PTA and cerebral aneurysms. To our knowledge, this is the first report describing an anterior inferior cerebellar artery (AICA) aneurysm in a patient with PTA.

## Case presentation

**Figure 1:**
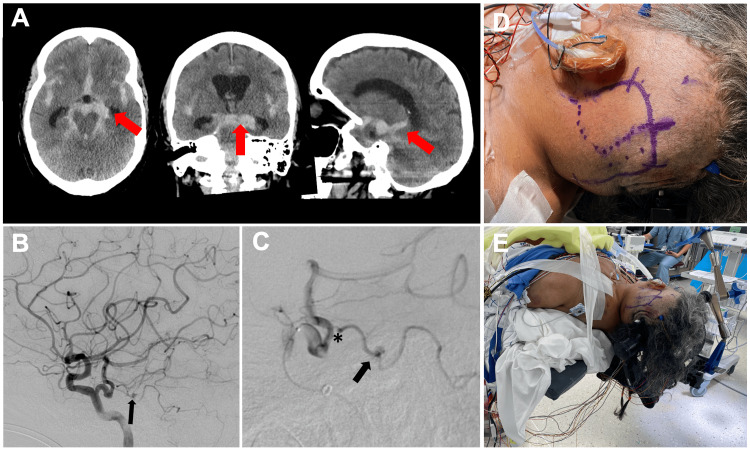
An 82-year-old female was found to have modified Fisher grade IV diffuse SAH on a head CT scan with IVH and obstructive hydrocephalus (A) Head CT scan in axial, coronal, and sagittal reconstruction indicating SAH located in the basal cisterns with IVH and communicating hydrocephalus (arrows). (B) Lateral left internal carotid artery angiogram showing a left Saltzman Type 1 PTA and a left AICA aneurysm (a2 segment) (arrow). (C) Selective PTA microcatheter injection showing the origin of AICA from PTA with origin stenosis (asterisk) and A2 aneurysm (arrow). (D) Hockey stick skin incision and marking the course of the left OA (dotted line) using navigation and Doppler for potential OA-AICA bypass. (E) Supine patient positioning with head turned for an extended retrosigmoid approach using a radiolucent head clamp for intraoperative angiogram. AICA, anterior inferior cerebellar artery; IVH, intraventricular hemorrhage; OA, occipital artery; PTA, persistent trigeminal artery; SAH, subarachnoid hemorrhage

**Figure 2:**
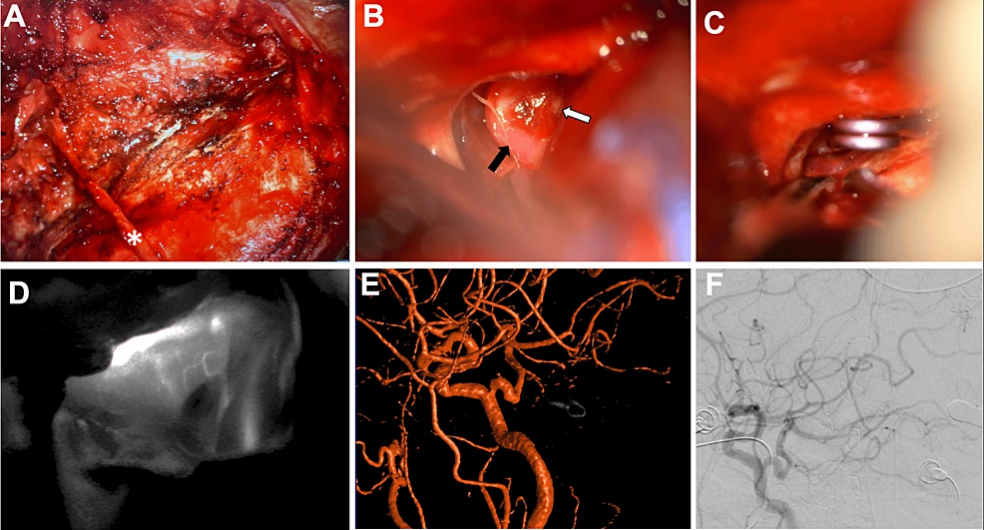
Conversion to open microsurgery through a left-sided extended retrosigmoid approach Intraoperative microscopic images (A) after OA harvest (asterisk) before craniotomy, (B) retrosigmoid exposure and visualization of distal AICA (black arrow) and aneurysm (white arrow), and (C) clipping of the aneurysm. (D) ICG angiography confirmed patent distal AICA, and intraoperative (E) three-dimensional and (F) two-dimensional left common carotid artery angiograms confirmed complete occlusion of the clipped aneurysm. AICA, anterior inferior cerebellar artery; ICG, immediate indocyanine green; OA, occipital artery

An 82-year-old female presented to the emergency department after being found obtunded at home and was found to have a modified Fisher grade IV diffuse subarachnoid hemorrhage on a head CT scan with intraventricular hemorrhage and obstructive hydrocephalus (Figure 1A) requiring external ventricular drain placement. Conventional angiography was notable for a multilobulated 2.2 × 1.9 mm AICA aneurysm (Figure 1B), which was thought to be the likely source of hemorrhage, concomitantly with a Saltzman Type 1 persistent left trigeminal artery. Endovascular treatment attempts failed due to the small diameter of the left AICA, stenosis of the vessel origin, and diminutive or tortuous course of the left AICA, which precludes selective catheterization, which is further complicated by the presence of PTA (Figures 1C, 1D, 1E). Therefore, the decision was made to proceed via open microsurgery through a left-sided extended retrosigmoid approach. In the first step, the left occipital artery was harvested for a potential extracranial-intracranial (EC-IC) as needed (Figure 2A). After microsurgical arachnoid dissection and exposure of the distal AICA, the vessel was followed back toward the BA, and the aneurysm came into view at the petrous apex (Figure 2B). The intraoperative rupture occurred during the final aneurysm dissection due to the fragile dome of the aneurysm, which was inaccessible. Due to the fragile and partially fusiform incorporation of the aneurysm with the parent vessel, primary clipping was unfeasible (Figure 2C). Immediate indocyanine green angiography showed rapid and instant distal collaterals to the AICA from superior cerebellar artery (SCA) and posterior inferior cerebellar artery (PICA), indicating adequate perfusion; hence, neither EC-IC nor intracranial-intracranial bypass were needed. A complete aneurysmal occlusion was confirmed with an intraoperative catheter angiogram (Figures 2D, 2E, 2F), and surgery was completed without intraoperative complications besides the intraoperative aneurysm rupture. The patient recovered well without postoperative deficits.

## Discussion

To the authors’ knowledge, this is the first reported case of AICA aneurysm in the setting of PTA. The initial endovascular treatment attempt failed due to the small caliber of AICA and origin stenosis precluding adequate access, as well as the tortuousness of the posterior cerebral vasculature, a known anatomical variation seen in PTA patients [14], necessitating open microsurgery. In line with prior reports [12], PTA-associated aneurysms are generally saccular and have a strong female predominance, which was the case here as well. However, the reported locations of aneurysms associated with PTA are almost exclusively in the anterior circulation, most commonly from PCOM [15], anterior cerebral artery [16], and middle cerebral artery [17], with only one literature report describing posterior circulation BA aneurysm with the presence of PTA, which was treated endovascularly [13]. The reasons behind the rarity of posterior circulation aneurysms, in particular, with PTA remain to be elucidated but may be explained by the different flow direction pattern; in the present case, for example, the PTA directed flow toward the AICA origin. Additionally, the small-caliber vertebrobasilar circulation and torturous PTA precluding successful endovascular treatment and necessitating open microsurgical intervention adds to the unique aspects of the above case.

The pathophysiology behind incomplete regression of the PTA remains poorly understood. Early embryonic development, starting at the 3-4 mm embryo stage (Carnegie stages 11 and 12), is characterized by the connection between the carotid arteries and the precursor to the vertebrobasilar circulatory system [1]. Four primitive anastomoses exist in total: trigeminal, otic, hypoglossal, and proatlantic intersegmental arteries; these transient vessels serve to temporarily supply oxygenated blood to the longitudinal neural artery, a forerunner of the vertebrobasilar artery, and eventually to the embryonic hindbrain. These anastomoses usually regress after approximately one week, when the posterior communicating and vertebrobasilar arteries have developed. Persistence of these primitive embryonic vessels can occur, most frequently PTA, with an estimated incidence of 0.5-0.7% [2-4]. The typical anatomic presentation of PTA has been studied in several cadaveric dissection case reports [18-20]. PTA typically originates from the posterolateral or posteromedial wall of the intracavernous interior carotid artery at the C4 segment and follows either a lateral (petrosal) or medial (sphenoidal) course by running lateral or medial to the abducens nerve, respectively, before piercing the dura. PTA proximity to the abducens nerve can cause neurologic deficits and, in severe cases such as a large aneurysm in the PTA trunk, a mass effect in the cavernous sinus with oculomotor and abducens nerve palsy [14]. The termination pattern of a PTA has also been classified by Saltzman into Type 1, featuring PTA termination in the BA between the AICA and SCA, causing BA and PCOM hypoplasia proximal to the site of anastomosis and Type 2, featuring PTA termination in the BA and supplying the SCA bilaterally, with the corresponding PCOM supplying the posterior cerebral artery [21]. Other anatomical variants have been noted, including PTA termination on the SCA, AICA, and PICA [22].

The bifurcating nature of the PTA initially heralded the belief that intracranial aneurysms had a significant association with PTA presence [14]. Indeed, initial studies placed the incidence of aneurysms in PTA patients as high as 14-32% [2,9]. However, the much lower incidence rates ranging from 3% to 4% in recent studies [3,10] with larger population samples indicate the initially exaggerated association between PTA and aneurysm formation. This may have been a result of selection bias in favor of patients undergoing cerebral angiography for existing symptomatic aneurysms. Nonetheless, PTA presence can complicate the endovascular treatment of pre-existing and otherwise unrelated aneurysms; therefore, having an adequate understanding of its supply territory is of paramount importance. In our case, the Saltzman Type 1 PTA installed itself in the BA between the AICA and SCA, causing the blood supply of arteries distal to the site of the anastomosis to depend entirely on the PTA. Furthermore, the diminutive diameter of the left AICA, along with the associated stenosis of the vessel origin and its tortuous course, precluded selective catheterization for aneurysmal embolization in this case, and thus microsurgery was indicated.

## Conclusions

We report a case of a ruptured AICA aneurysm in the setting of PTA, treated with open microsurgical trapping after the failure of an endovascular treatment attempt. Posterior circulation aneurysms associated with PTA are extremely rare, which might be related to its underdevelopment or hypoplasia in PTA presence. These subsequent anomalies might render endovascular treatment challenging. Therefore, having an adequate understanding of the PTA impact on cerebral vasculature is of paramount importance, and open microsurgical treatment is important to keep in mind.
